# Blood spots as an alternative to whole blood collection and the effect of a small monetary incentive to increase participation in genetic association studies

**DOI:** 10.1186/1471-2288-9-76

**Published:** 2009-11-13

**Authors:** Parveen Bhatti, Diane Kampa, Bruce H Alexander, Christopher McClure, Danny Ringer, Michele M Doody, Alice J Sigurdson

**Affiliations:** 1 Program in Epidemiology, Division of Public Health Sciences, Fred Hutchinson Cancer Research Center, Seattle, WA, USA; 2 Radiation Epidemiology Branch, Division of Cancer Epidemiology and Genetics, National Cancer Institute, NIH, DHHS, Bethesda, MD, USA; 3 Division of Environmental Health Sciences, School of Public Health, University of Minnesota, Minneapolis, MN, USA; 4 Research Triangle Institute, Rockville, MD, USA

## Abstract

**Background:**

Collection of buccal cells from saliva for DNA extraction offers a less invasive and convenient alternative to venipuncture blood collection that may increase participation in genetic epidemiologic studies. However, dried blood spot collection, which is also a convenient method, offers a means of collecting peripheral blood samples from which analytes in addition to DNA can be obtained.

**Methods:**

To determine if offering blood spot collection would increase participation in genetic epidemiologic studies, we conducted a study of collecting dried blood spot cards by mail from a sample of female cancer cases (n = 134) and controls (n = 256) who were previously selected for a breast cancer genetics study and declined to provide a venipuncture blood sample. Participants were also randomized to receive either a $2.00 bill or no incentive with the blood spot collection kits.

**Results:**

The average time between the venipuncture sample refusal and recruitment for the blood spot collection was 4.4 years. Thirty-seven percent of cases and 28% of controls provided a dried blood spot card. While the incentive was not associated with participation among controls (29% for $2.00 incentive vs. 26% for no incentive, p = 0.6), it was significantly associated with participation among the breast cancer cases (48% vs. 27%, respectively, p = 0.01). There did not appear to be any bias in response since no differences between cases and controls and incentive groups were observed when examining several demographic, work history and radiation exposure variables.

**Conclusion:**

This study demonstrates that collection of dried blood spot cards in addition to venipuncture blood samples may be a feasible method to increase participation in genetic case-control studies.

## Background

Increasingly, large epidemiologic cohort and case-control studies include collection of biologic specimens because genetic and/or clinical information are key elements of the hypotheses under study. Many studies have collected buccal cells from saliva for DNA extraction; however, blood samples are more versatile because in addition to DNA, several analytes can be measured in the serum [1]. Venipuncture blood collection can be difficult to undertake due to cost, organizational complexity, and shipping and storage requirements. More importantly, subject inconvenience (i.e. arranging an appointment with and travelling to see a phlebotomist) and invasiveness of the method can decrease participation. Alternatively, dried blood spot collection of capillary blood via finger stick offers an economical, easily transportable and storable means of collecting peripheral blood samples for high quality DNA and numerous other biomarkers [1-7]. As compared to venipuncture, blood spot collection is more convenient (i.e. can be done by oneself at home), and while this method is invasive, it may be considered less so among participants. In the U.S. Radiologic Technologists (USRT) study, we have routinely collected venipuncture blood samples, but cost concerns as well as moderate response proportions prompted an evaluation of alternative methods for collecting peripheral blood samples.

To determine if offering blood spot collection would increase participation in genetic epidemiologic studies, we conducted a study of collecting dried blood spot cards by mail among 390 female USRT cohort participants selected for a nested case-control study of breast cancer who declined to provide a venipuncture blood sample. We also assessed the impact of an incentive to encourage participation by randomizing participants to receive either a $2.00 bill or no incentive with the blood spot collection kits.

## Methods

This study was approved by the human subjects review boards of the National Cancer Institute and the University of Minnesota.

### Study population

Study participants were women that were selected from eligible cases and controls identified as part of a genetic case-control study of breast cancer (between 1999 and 2003) nested in the USRT cohort [8] who had declined to provide a venipuncture blood sample. Of 1402 eligible breast cancer cases, 871 (62%) agreed to participate in the original study. One-hundred and fifty-five cases could not be located or were unable to participate, and 376 refused to participate, of which 343 specifically declined to provide a venipuncture blood sample. Of the 2,268 breast cancer controls that were identified, 1093 (48%) agreed to participate in the genetics study. Three-hundred and thirty-six controls could not be located or were unable to participate; 839 controls refused to participate in the study of which 814 specifically declined to provide a venipuncture blood sample.

Of the 343 cases and 814 controls, subjects were excluded from the blood spot study if they began working before 1950 (n = 171), did not complete the most recent cohort survey (n = 349), gave blood for an earlier genetic study (n = 9), previously refused any DNA collection (n = 31), were deceased (n = 55), too ill or unable to participate (n = 6), taking blood thinners (n = 5) or had withdrawn from further study participation (n = 29). During 2006, all remaining cases (n = 134) and a random sample of controls (n = 256) were recruited. The time between refusal to provide a venipuncture sample and recruitment for blood spot collection ranged from 2 to 9 years (mean = 4.4). Within case and control groups, subjects were randomized for contact order and then for receipt of a $2.00 bill incentive with the blood spot collection kit. Each technologist was sent an advance letter by the US postal system, followed one week later by a blood spot collection kit, with up to 3 follow-up mailings or telephone calls for non-responders approximately every 3 weeks. Sixty-seven cases and 129 controls received no incentive, and 67 cases and 127 controls received the incentive. Demographic variables for these subjects were obtained from previously administered questionnaires [9]; reconstruction of occupational ionizing radiation breast doses and personal diagnostic ionizing radiation exposures has been described previously [8].

### Dried blood spot kit

Each subject was mailed a self-collection kit that contained a dried blood spot collection card (Whatman Protein Saver Card for five 75-80 μL blood samples), a BD Genie™ lancet (2.0 mm depth, 1.5 mm width), alcohol wipe, gauze pad, adhesive bandage, desiccant pouch, and a foil bag for the completed blood spot card. A consent form, detailed instructions for self-collection of a finger stick capillary dried blood spot sample and a questionnaire to collect updated breast cancer risk factor information were included with the kit, along with an envelope and first class mailing stamp for the subjects to return the specimens.

### Statistical analysis

We used contingency table and logistic regression analyses (SAS Institute, Cary, North Carolina, Release 8.02) to examine participation in the dried blood spot collection according to incentive, time between initial refusal and the request for dried blood spots, radiation exposure and demographic characteristics including year of birth, marital status, smoking status and region of residence. We also used t-tests to compare log-transformed occupational breast radiation dose and personal diagnostic radiation breast exposure scores between participants and non-participants. Analyses were stratified by case-control status. To assess whether the incentive and other characteristics differentially affected participation among cases and controls, we added cross products terms to logistic regression models that adjusted for the main effect of case-control status and the variable under consideration. Because 95% of our subjects were non-Hispanic Caucasian, we did not examine associations with race/ethnicity; however, the results did not differ when we restricted analyses to non-Hispanic Caucasian subjects. Mutual adjustment for the variables evaluated in this study had minimal impact on the point estimates of interest (< 10%) in our logistic regression models, so we present univariate results only, except for the radiation exposure variables, for which the analyses were adjusted for year of birth because of the potential confounding effects of age; the greater the age, the more opportunity to be exposed to occupational and personal diagnostic radiation. All statistical tests were two-sided and statistical significance was assessed at p ≤ 0.05.

## Results

Of 390 cases and controls in the USRT breast cancer genetic study who previously declined to provide a venipuncture blood sample, 121 (31%) provided a dried blood spot card. A greater proportion of cases participated in the blood spot collection than controls (37% versus 28%) (p = 0.07).

Cases who received the $2.00 bill were more likely to provide a dried blood spot card than were cases who received no incentive (48% versus 27%; OR = 2.5; 95% CI: 1.2-5.1; p = 0.01) (Table 1); however we found little difference in control participation according to incentive (29% for the $2.00 bill versus 26% for no incentive; OR = 1.1; 9.5% CI: 0.7-2.0; p = 0.6). The test for a differential effect of incentive on participation between cases and controls resulted in a p-value of 0.09. On average, a greater number of years passed between collection efforts for cases (mean = 5.5 years) than for controls (mean = 3.7 years). There was some indication in Table 1 that cases were more likely and controls were less likely to participate as a greater number of years passed between collection efforts. These results, however, were based on small numbers in the extreme categories and were not statistically significant. Furthermore, controlling for years between collection efforts in logistic regression models assessing the impact of incentive had little impact on the point estimates.

**Table 1:** Dried blood spot collection among breast cancer cases and control who initially declined to provide a venipuncture blood sample, according to incentive, demographic characteristics and selected cancer risk factors

	Cases (n = 134)				Controls (n = 256)			
	No blood spot	Blood spot	OR (95% CI)	p-value^a^	No blood spot	Blood spot	OR (95% CI)	p-value^a^
Incentive Group								
No incentive	49 (73%)	18 (27%)	ref	0.01	95 (74%)	34 (26%)	ref	0.6
$2 incentive	35 (52%)	32 (48%)	2.5^b^(1.2,5.1)		90 (71%)	37 (29%)	1.1 (0.7,2.0)	
Year of birth								
< 1935	18 (56%)	14 (44%)	ref	0.8	32 (63%)	19 (37%)	ref	0.4
1935 to 1944	35 (63%)	21 (38%)	0.8 (0.3,1.9)		90 (75%)	30 (25%)	0.6 (0.3,1.1)	
1945 to 1954	24 (67%)	12 (33%)	0.6 (0.2,1.7)		53 (29%)	19 (26%)	0.6 (0.3,1.3)	
≥ 1955	7 (70%)	3 (30%)	0.6 (0.1,2.5)		10 (77%)	3 (23%)	0.5 (0.1,2.0)	
Marital Status								
Married/Living Together	53 (61%)	34 (39%)	ref	0.8	135 (73%)	50 (27%)	ref	0.9
Widowed/ Divorced/ Seperated	23 (66%)	12 (34%)	1.1 (0.6,2.2)		38 (70%)	16 (30%)	1.1 (0.6,2.2)	
Never Married	8 (67%)	4 (33%)	1.1 (0.4,3.4)		12 (71%)	5 (29%)	1.1 (0.4,3.3)	
Smoking Status								
Never	39 (56%)	31 (44%)	ref	0.2	99 (71%)	40 (29%)	ref	0.8
Former	36 (71%)	15 (29%)	0.5 (0.2,1.1)		60 (75%)	20 (25%)	0.8 (0.4,1.5)	
Current	9 (69%)	4 (31%)	0.6 (0.6,2.0)		26 (72%)	10 (28%)	1.0 (0.4,2.2)	
Unknown	0 (0%)	0 (0%)	N/A		0 (0%)	1(100%)	N/A	
Region of residence^c^								
Northeast	16 (57%)	12 (43%)	ref	0.7	29 (67%)	14 (33%)	ref	0.9
Midwest	19 (63%)	11 (37%)	0.8 (0.3,2.2)		48 (73%)	18 (27%)	0.8 (0.3,1.8)	
South	15 (54%)	13 (46%)	1.2 (0.4,3.3)		37 (69%)	17 (31%)	1.0 (0.4,2.2)	
West	11 (61%)	7 (39%)	0.8 (0.2,2.7)		24 (69%)	11 (31%)	0.9 (0.4,2.5)	
Unknown	23 (77%)	7 (23%)	0.4 (0.1,1.2)		47 (81%)	11 (19%)	0.5 (0.2,1.2)	
Occupational radiation breast dose (Gy)								
0 to 0.02	56 (65%)	30 (35%)	ref	0.9	124 (72%)	49 (28%)	ref	0.02
> 0.02 to 0.04	13 (59%)	9 (41%)	1.2 (0.5,3.4)		34 (89%)	4 (11%)	0.3 (0.1,0.9)	
> 0.04 to 0.06	6 (60%)	4 (40%)	1.2 (0.3,4.8)		10 (71%)	4 (29%)	1.0 (0.3,3.4)	
> 0.06	9 (56%)	7 (44%)	1.5 (0.5,4.3)		17 (55%)	14 (45%)	2.1 (0.9,4.6)	
Personal diagnostic radiation breast dose score								
0 to 0.02	47 (67%)	23 (33%)		0.5	121 (73%)	44 (27%)		0.9
> 0.02 to 0.04	12 (50%)	12 (50%)	2.0 (0.8,5.2)		33 (70%)	14 (30%)	1.2 (0.6,2.4)	
> 0.04 to 0.06	9 (60%)	6 (40%)	1.4 (0.4,4.3)		14 (74%)	5 (26%)	1.0 (0.3,2.9)	
> 0.06	16 (64%)	9 (36%)	1.1 (0.4,3.0)		17 (68%)	8 (32%)	1.3 (0.5,3.2)	

^a^Chi-square test excluded Unknown category

^b^p-value for super-multiplicative effect modification = 0.09

^c^U.S. Census Bureau definition: Northeast = CT, ME, MA, NH, RI, VT, NJ, NY, PA, DE, DC, MD; Midwest = IL, IN, MI, OH, WI, IA, KS, MN, MO, NE, ND, SD; South = FL, GA, NC, SC, VA, WV, AL, KY, MS, TN, AR, LA, OK, TX; West = AZ, CO, ID, MT, NV, NM, UT, WY, AK, CA, HI, OR, WA

After controlling for year of birth, we also observed a borderline significant difference in the distribution of occupational radiation breast dose among controls who did and did not provide a dried blood spot card, with a greater proportion of participating compared to non-participating controls being in the highest radiation dose category (> 0.06 Gy) (Table 1). However, there was no significant difference between the means of the log-transformed occupational radiation breast doses among control participants (mean = 0.02 Gy) and non-participants (mean = 0.03 Gy) (p = 0.5). We observed no significant differences in participation among cases and controls according to the other variables listed in Table 1, but there was a tendency for younger individuals among cases and controls and former and current smokers among cases to be less likely to participate

## Discussion

This study demonstrates that collection of dried blood spot cards in addition to venipuncture blood samples may be a feasible method to increase overall participation in genetic case-control studies. Among randomly selected women who previously declined to provide a venipuncture blood sample for a breast cancer case-control study [8], dried blood spot cards were successfully collected from 37% of cases and 28% of controls. For the underlying breast cancer case-control study [8], assuming that no incentive was offered and applying a conservative response estimate of 25%, we estimate that collection of dried blood spots after an initial attempt to collect venipuncture blood samples would increase the overall participation among cases from 62% to 68% and among controls from 48% to 57%.

Convenience of blood spot collection over venipuncture blood draw is the most likely reason for the increase in participation. Subjects were able to perform the procedure at home and return the blood spot card through regular mail. In comparison, venipuncture necessitated scheduling appointments at laboratories or clinics and then arranging to have the blood samples picked up by a courier service. In general, the collection of capillary blood by finger stick is considered to be less invasive than blood draw using venipuncture because there is lower risk of soft tissue injury. However, there may have been discomfort and difficulties in applying the finger stick procedure among study subjects, so we can only speculate as to whether relative invasiveness was a factor in increased participation.

Our results suggest a differential effect of the $2.00 incentive between cases and controls, with cases responding more favorably to the incentive. We compared demographic characteristics between cases and controls and did not find any significant differences to explain the increased response among cases. A differential response proportion among cases and controls raises the concern of potential response bias, although we did not find any significant differences when comparing characteristics between the cases who responded to the $2.00 bill and the cases who did not.

We also observed a significant difference in the distribution of occupational radiation breast doses between controls who provided a blood spot card and controls who did not. It appeared that a greater proportion of controls with higher doses chose to participate, however, this finding was based on a small number of technologists in the highest dose category, and the difference in the log transformed mean occupational ionizing radiation breast doses between the participating and non-participating controls was not significant.

The use of financial incentives to improve response to questionnaires has been previously investigated in the USRT cohort [10]; the response proportion for the $2.00 bill among persistent non-responders to a questionnaire survey was similar to the response proportion we observed among controls receiving the $2.00 bill in the present study (29%). Coogan et al examined the effect of financial incentives on case and control participation in a telephone interview for a study of colorectal cancer [11]. They found that cases randomized to receive $5.00 were slightly less likely to participate than cases that were not offered the incentive (64% and 68%, respectively). However, participation among controls offered a $5.00 incentive was significantly higher than among controls approached for participation in the previous year without an incentive (56 and 44% respectively, p < 0.001). Gilbart et al and Parkes et al found that $5.00 incentives increased responses to questionnaires among controls from cancer case-control studies by 20 and 15%, respectively [12,13]; Parkes et al [8] also found that a $2.00 bill increased participation among controls by 11%. The focus of previous incentive studies was questionnaire response, so strict comparison of response to blood collection in the present study is difficult.

Strengths of the present study include the examination of incentives among both cases and controls and the ability to examine the effect of a variety of factors on participation, including primary exposure variables which are unavailable for non-responders in many studies. We did not observe any significant differences among characteristics that we evaluated; however some comparisons may have had limited power because of small numbers of technologists in the comparison groups.

In this study, two to nine years passed between the collection efforts. It is possible that blood spot recruitment following more immediately after requesting a venipuncture sample or offered in lieu of a venipuncture sample may have affected our response proportions. Nonetheless, the length of this time period was not a significant predictor of participation and adjustment for the time between collection efforts had a minimal effect on our results.

The applicability of our results to other populations, particularly men, is another limitation of this study given that participants were women that worked as medical professionals and were predominantly non-Hispanic. Furthermore, we were unable to assess the quantity or quality of the genetic material obtained from the blood spot cards collected for this study; however, it has been previously demonstrated that blood spots are a stable source of high quality DNA [1] that can be effectively used to conduct genome-wide association studies [3].

## Conclusion

While the generalizability of our findings are limited due to the focus on female radiologic technologists and the time period between biospecimen collection efforts, our study does demonstrate that dried blood spots may be a feasible method for increasing participation in genetic studies. Other genetic or biomarker studies may choose to consider collecting dried blood spot cards from study subjects given their convenience, ease of transport and storage, versatility and lack of demonstrable bias by case-control status or incentive.

## Competing interests

The authors declare that they have no competing interests.

## Authors' contributions

PB performed the statistical analysis and drafted the manuscript. DK, BH, CM and DR participated in the design and coordination of the study. MD and AS conceived of the study, participated in its design and coordination and, all authors contributed to early and final manuscript drafts.

## Pre-publication history

The pre-publication history for this paper can be accessed here:

http://www.biomedcentral.com/1471-2288/9/76/prepub
